# Select Skeletal Muscle mRNAs Related to Exercise Adaptation Are Minimally Affected by Different Pre-exercise Meals that Differ in Macronutrient Profile

**DOI:** 10.3389/fphys.2018.00028

**Published:** 2018-01-26

**Authors:** Pim Knuiman, Maria T. E. Hopman, Jeroen A. Wouters, Marco Mensink

**Affiliations:** ^1^Division of Human Nutrition, Wageningen University and Research, Wageningen, Netherlands; ^2^Department of Physiology, Radboud University Medical Centre, Nijmegen, Netherlands; ^3^Centre for Sporting Excellence and Education, Sportcentre Papendal, Arnhem, Netherlands

**Keywords:** nutrient availability, endurance exercise, resistance exercise, gene expression, humans

## Abstract

**Background:** Substantial research has been done on the impact of carbohydrate and fat availability on endurance exercise adaptation, though its role in the acute adaptive response to resistance exercise has yet to be fully characterized.

**Purpose:** We aimed to assess the effects of a pre-resistance exercise isocaloric mixed meal containing different amounts of carbohydrates and fat, on post-resistance exercise gene expression associated with muscle adaptation.

**Methods:** Thirteen young (age 21.2 ± 1.6 year), recreationally trained (VO_2max_ 51.3 ± 4.8 ml/kg/min) men undertook an aerobic exercise session of 90-min continuous cycling (70% VO_2max_) in the morning with pre- and post-exercise protein ingestion (10 and 15 g casein in a 500 ml beverage pre- and post-exercise, respectively). Subjects then rested for 2 h and were provided with a meal consisting of either 3207 kJ; 52 g protein; 51 g fat; and 23 g carbohydrate (FAT) or 3124 kJ; 53 g protein; 9 g fat; and 109 g carbohydrate (CHO). Two hours after the meal, subjects completed 5 × 8 repetitions (80% 1-RM) for both bilateral leg press and leg extension directly followed by 25 g of whey protein (500 ml beverage). Muscle biopsies were obtained from the *vastus lateralis* at baseline (morning) and 1 and 3 h post-resistance exercise (afternoon) to determine intramuscular mRNA response.

**Results:** Muscle glycogen levels were significantly decreased post-resistance exercise, without any differences between conditions. Plasma free fatty acids increased significantly after the mixed meal in the FAT condition, while glucose and insulin were higher in the CHO condition. However, PDK4 mRNA quantity was significantly higher in the FAT condition at 3 h post-resistance exercise compared to CHO. HBEGF, INSIG1, MAFbx, MURF1, SIRT1, and myostatin responded solely as a result of exercise without any differences between the CHO and FAT group. FOXO3A, IGF-1, PGC-1α, and VCP expression levels remained unchanged over the course of the day.

**Conclusion:** We conclude that mRNA quantity associated with muscle adaptation after resistance exercise is not affected by a difference in pre-exercise nutrient availability. PDK4 was differentially expressed between CHO and FAT groups, suggesting a potential shift toward fat oxidation and reduced glucose oxidation in the FAT group.

## Introduction

The fundamental phenotypic adaptation to repeated bouts of resistance exercise is an increase in muscle mass and strength. It is assumed that the cumulative effect of frequent resistance exercise sessions within a certain period of time modifies specific proteins, thereby enabling or enhancing the crucial biological processes required for adaptation (Pilegaard et al., 2000; Flück et al., 2005; Coffey et al., 2009). Considerable attention has been paid to the time course of mRNA response in relation to exercise, and we now know that the exercise-induced changes in mRNA quantity peak immediately post-exercise up to 12 h and return to basal levels within 24 h (Bickel et al., 2005; Mahoney et al., 2005; Yang et al., 2005; Louis et al., 2007).

It is well-known that manipulating nutrition affects the acute intramuscular response with exercise. For instance, post-resistance exercise protein provision augments the AKT-mTOR-S6K signaling pathway to initiate translation (Glass, 2005; Baar, 2014). On the other hand, carbohydrate restriction with endurance exercise reduces muscle glycogen levels, which in turn affect mRNA levels of genes involved in mitochondrial biogenesis (Bartlett et al., 2015; Impey et al., 2016). In addition, undertaking resistance exercise with low skeletal muscle glycogen levels enhances PGC-1α mRNA quantity (Camera et al., 2015). The mRNA response to resistance exercise has been examined under a variety of nutritional states (fasted vs. fed; protein vs. placebo) (Coffey et al., 2009; West et al., 2011), however, the role of pre-exercise fat and carbohydrate availability on the acute adaptive response remains to be examined. Additionally, most literature assessing low-carbohydrate diets (Burke and Hawley, 2002; Burke and Kiens, 2006; Noakes et al., 2014; Burke, 2015; Volek et al., 2015) or low-carbohydrate availability with exercise (Morton et al., 2009; Hulston et al., 2010; Cochran et al., 2015; Marquet et al., 2016) has focused on endurance exercise. Consequently, specific guidelines for daily carbohydrate intake and timing regarding resistance exercise training are primarily derived from endurance models and the available data on the acute resistance exercise response with different carbohydrate availability. However, to our knowledge, it remains unclear whether pre-resistance exercise carbohydrate or fat availability affects the post-resistance exercise mRNA response when matched for protein intake. Therefore, we aimed to explore the effects of differences in carbohydrates and fat availability on post-resistance exercise gene expression. After a glycogen depleting endurance exercise session in the morning subjects received an isocaloric mixed meal containing different amounts of carbohydrates and fat 2 h before a resistance exercise session in the afternoon, while ample protein was provided throughout the day. We hypothesize that some of the post-resistance exercise selected mRNAs e.g., PDK4, PGC-1α, and SIRT1 will respond differently to the nutritional conditions, without any changes in proteolytic genes.

## Methods

### Subject characteristics

All subjects were non-smokers, free of injury, and not using any medication or nutritional supplements. All subjects provided a full-written informed consent. This study was carried out in accordance with the guidelines for human research of The Medical Ethical Committee of Wageningen University. The Medical Ethical Committee of Wageningen University approved all study procedures. Fourteen healthy physically-active males volunteered to participate in this study: age 21.2 ± 1.6 years, height 1.87 ± 0.05 cm, weight 76.7 ± 4.7 kg, leg extension 1-RM 111 ± 11 kg, leg press 1-RM 266 ± 30 kg and VO_2max_ 51.3 ± 4.8 ml/kg/min^−1^ (see Table 2). One subject dropped out after test day 1 because of discomfort of the muscle biopsies. Physical characteristics of 13 volunteers are shown in Table 2.

### Study design

**Figure 1:**
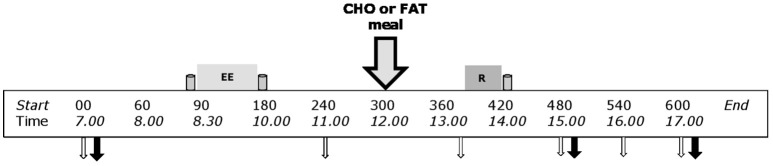
Schematic diagram of the experimental trials. This study adopted a counterbalanced crossover design where subjects completed both exercise trials with different nutritional treatments on separate occasions. Endurance exercise (EE) session: 90-min cycling at 70% VO_2max_. Resistance exercise (R) sessions 5 sets of 8 repetitions for both leg press and leg extensions at 80% 1-RM. Gray containers indicates protein beverage (from left to right; 10 g casein, 15 g casein, and 25 g whey). Big gray arrow with black outline indicates lunch with carbohydrates or fat (nutritional intervention meal). Open white arrows indicates time point for blood sample; black arrow indicates sampling time point for muscle biopsy.

This study used a crossover design with two experimental days. On both experimental days subjects completed the same protocol: an endurance exercise session in the morning (08.30–10.00 a.m.) and a resistance exercise session in the afternoon (02.00–02.30 p.m.) with a resting period of 4 h between sessions (Figure 1). The subjects received a mixed meal in between the exercise sessions (12.00 p.m.), which was either a high carbohydrate—low fat meal (CHO) or a low carbohydrate—high fat meal (FAT). Each trial was separated by a minimum of 12 days (range: 12–30 days), during which time the subjects were instructed to maintain their habitual lifestyle.

### Preliminary testing

#### Maximal aerobic capacity (VO_2max_)

Preliminary testing was performed in the week prior to the start of the first experimental day. Following a 30-min rest, subjects performed a ramped VO_2max_ test on an electrically braked cycle ergometer (Lode Excalibur, Groningen, the Netherlands). After a 5-min warm up at 50 W, the subjects started cycling at 100 W. Workload was progressively increased by 20 W·min^−1^ until the subject reached volitional exhaustion. The VO_2max_ test was considered to be valid when two out of three criteria were met: (I) leveling of VO_2_ with increasing workload; (II) heart rate within 10 beats of the theoretically estimated maximum (220-age); and (III) respiratory exchange ratio (RER) of ≥1.15. Oxygen consumption (VO_2_) was measured through breath-by-breath sampling with an Oxycon Pro (Jaeger, Hoechberg, Germany) to define maximal oxygen consumption (VO_2max_). Subjects were asked to maintain a cadence between 80 and 100 r·min^−1^.

#### Maximal strength (1RM)

Subjects were asked to arrive fasted (at least 4 h) and to avoid any strenuous physical activity 48 h before the preliminary tests. Each subject performed a bilateral one-repetition maximum (1RM) using the leg press and leg extension machine (Technogym, Cesena, Italy). Subjects were familiarized with the movement and warmed up prior to testing by performing six repetitions (at ~40% of estimated 1RM) through a full range of motion with a 1-min rest. After each successful lift, the weight was increased until a failed attempt occurred with 3-min recovery between each attempt. The 1RM was attained within five attempts. Subjects performed a familiarization session for both the endurance and the resistance exercise session ~1 week before the first experimental day.

### Diet and physical activity control

Subjects were instructed to avoid alcohol, caffeine and physical activity during 48 h prior to the 2 experimental days. Diet and physical activity levels were recorded for 24 h before the first experimental day and subjects were asked to replicate dietary intake and physical activity prior to the second experimental day. A standardized meal for the subjects was provided the night before each trial (standard deep-frozen meal and ice-cream dessert; 43.80 kJ/kg BW; 15 Energy% protein, 30 Energy% fat, and 55 Energy% carbohydrate).

### Experimental days

All subjects arrived in the laboratory at 07.00 being fasted since 10 p.m. the evening before. After changing into sport gear, a catheter was inserted into the antecubital vein of the left arm. Fifteen minutes after insertion of the catheter baseline blood samples were taken followed by a baseline muscle biopsy. Approximately 10 min after the baseline biopsy a protein beverage containing 10 g of casein dissolved in 350 ml water was consumed (*t* = −05 min). Within 5 min after consumption of the protein beverage, the subjects started with 90 min cycling (*t* = 0 min) at submaximal intensity (70% VO_2max_). If subjects could not sustain the intensity of at least 60 RPM, resistance was decreased with 20 W, and the same intensity protocol was repeated during the second training. All subjects performed same exercise intensity of both experimental days. Immediately after the endurance exercise bout, a beverage containing 15 gram of casein dissolved in 350 ml water was consumed (*t* = 90 min). A second blood sample (~25 ml) was taken 1 h after the end of the endurance exercise bout (*t* = 240 min). Two hours after the endurance exercise bout (*t* = 300 min) subjects were randomly provided with either a carbohydrate rich low-fat meal or low-carbohydrate fat meal (see intervention meal for details). Ninety minutes after the meal (*t* = 390 min) a third blood sample (~25 ml) was taken. At *t* = 390 min subjects performed the resistance exercise bout that consisted of 5 × 8 (80% 1RM) repetitions of bilateral leg press and leg extension with 2 min of rest in between the sets. If subjects could not sustain due to fatigue, the selected weight was decreased with steps of 10 or 5 kg for the leg press and leg extension, respectively. Immediately after the resistance exercise bout (*t* = 420 min), a protein beverage containing 25 g of whey protein dissolved in 350 ml water was consumed. One and three hours after the resistance exercise bout a second (*t* = 480 min) and a third (*t* = 600 min) skeletal muscle biopsy was taken. All biopsies were taken from the same leg. Additional blood samples were taken at *t* = 480, 540, and 600 min. A timeline for the experimental day can be found in Figure 1. The whole experimental protocol was repeated on the 2nd day, while the other meal was provided.

### Nutritional intervention meal

Both meals were prepared by a research dietician of Wageningen University. Both the carbohydrate and fat meal consisted of commercially available meat, macaroni, and vegetables with an energetic value of ~3200 kJ. The absolute macronutrient amounts can be found in Table 1.

**Table 1:** Physical characteristics of subjects.

	**Mean ± SE**
Age (years)	21.2 ± 0.5
Height (m)	1.87 ± 0.0
Weight (kg)	76.7 ± 1.3
BMI (kg/m^2^)	22.0 ± 0.2
W_max_ (W)	346 ± 7.6
VO_2max_ (ml/kg/min^−1^)	51.3 ± 1.3
Leg press 1RM (kg)	266 ± 8.2
Leg extension 1RM (kg)	111 ± 3.0

**Table 2:** Overview of the energy and macronutrient composition intervention meal.

**Energy & nutrient**	**Fat meal**	**Carbohydrate meal**
Energy (kJ)	3207	3124
Protein (g)	52	52
Fat (g)	51	9
Carbohydrates (g)	20	110

### Nutritional strategy

Although the findings on gene expression with exercise and carbohydrate restriction are inconsistent, carbohydrate restriction during and after endurance exercise may upregulate genes involved in mitochondrial biogenesis (Margolis and Pasiakos, 2013). Therefore, we decided to provide our subjects ~2 h post-exercise with carbohydrates. The protein supplementation strategy used in our study was mainly based on muscle protein synthetic response findings after exercise. A substantial body of research suggests that post-resistance exercise whey protein increases myofibrillar protein synthesis to a higher extend when compared with casein (Morton et al., 2015). In contrast, little evidence exist for the optimal type of protein to maximize the mitochondrial protein synthetic response after endurance exercise. We therefore decided to provide our subjects with a slow digestible casein protein before and after the endurance exercise sessions. Our nutritional strategy has been recently proposed by others (Perez-Schindler et al., 2015).

### Muscle biopsies

Muscle biopsies were taken as described by Bergstrom (1975). Biopsies were taken under local anesthesia (2–3 ml of 2% Xylocaine) using a 5-mm Bergstrom needle modified with suction. All three muscle biopsies per experimental day were taken from the vastus lateralis of the same leg, with separate incisions (~1–1.5 cm) apart and from distal to proximal direction. Muscle biopsies on the second test day were taken from the contralateral leg. Muscle biopsies were immediately frozen (in 5–10 s) in liquid nitrogen and stored at −80°C for subsequent biochemical analysis, after being freed from visible fat, blood, and connective tissue.

### Muscle glycogen

Muscle tissue, ~30 mg wet weight, was freeze dried, after which collagen, blood, and non–muscle fiber materials were removed from the muscle fibers under a microscope. The isolated muscle fiber mass (~5–7 mg) was weighed, and 500 μl of 1 M HCl was added. After heating for 3 h at 100°C to hydrolyze the glycogen to glycosyl units and cooling down to room temperature, 500 μl of the solution was neutralized by adding 280 μl of Tris–KOH (Tris 119 mM, KOH 2.14 M) and centrifuged at 1000 g and 4°C for 10 min. Thereafter, 150 μl of this solution was analyzed for glucose concentration (Glucose HK CP A11A01667, ABX Pentra) with a COBAS FARA semiautomatic analyzer (Roche).

### Blood samples and analyses

All blood samples were immediately centrifuged at 1000 g at 4°C for 10 min, after which plasma was stored in −80°C until further analysis. Blood samples were analyzed for cortisol, creatine kinase, glucose, and insulin (Gelderse Vallei hospital, Ede, NL). Cortisol was measured with immunometric chemilumiescence (sandwich) assay with Immulite XPi, (Siemens, the Netherlands). Creatine kinase was measured using Vista device (Siemens, the Netherlands). Free fatty acids were assessed using an enzymatic test kit according to the manufacturer's protocol (InstruChemie, Delfzijl, Netherlands). Glucose was measured using absorption changes caused by the formation of NADH as a measure of glucose concentration and was measured bichromatic (340 and 383 nm) by means of an end point technique (Siemens, the Netherlands). Insulin was measured using an solid-phase enzyme-linked chemilumiescent immunometric assay with Immulite 2000 XPi (Siemens, the Netherlands).

### RNA extraction and real-time quantitative PCR

For RNA isolation, muscle samples (27.8 ± 13.5 mg) were homogenized using an Ultra Turrax (Qiagen, Venlo, Netherlands, cat no: 9001272) and TRIzol-based kit according to manufacturer's guidelines (Fisher Thermo Scientific, Amsterdam, the Netherlands, cat no: 15596026). After homogenization in TRIzol, RNA isolation was performed using an RNeasy micro-kit according to the manufacturer's guidelines (Qiagen, Venlo, Netherlands, cat no: 74004). Briefly, chloroform was added to homogenized samples before centrifuging for 15 min at 10.000 rpm. Afterwards the aqueous phase was transferred to the RNeasy micro column and was washed for several times to isolate all RNA. Yield was quantified with a NanoDrop ND 1000 spectrophotometer (Nanodrop Technologies, Wilmington, United States of America), and integrity was measured with an Agilent 2100 Bioanalyzer with RNA 6000 Nano chips (Agilent Technologies, South Queensferry, United Kingdom).

### cDNA synthesis and real-time quantitative polymerase chain reaction

RNA from all samples was reversely transcribed to cDNA using a Fermentas cDNA synthesis kit according to the manufacturer's protocol (Fischer Thermo Scientific, Amsterdam, the Netherlands, cat no: K1612). Briefly, 500 ng of isolated RNA was diluted in 10 μl of RNase-free water and afterwards a mix for cDNA synthesis was added. When cDNA was formed, quantitative real-time polymerase chain reaction (qPCR) was conducted using SYBR Green on a Bio-Rad CFX384 machine (Bio-Rad Laboratories BV, Veenendaal, Netherlands). SensiMix (Bioline, London, United Kingdom) was used to carry out the PCR reaction. Primers for all genes, obtained from the Harvard PrimerBank (Spandidos et al., 2010), are shown in Table 3. Samples were run in duplicate, and all samples from each subject were run on the same plate to allow direct relative comparisons. Relative changes in mRNA expression for the genes of interest were quantified using the relative standard curve method (Larionov et al., 2005). qPCR data were normalized using GAPDH as housekeeping gene for the human samples, since it has shown to be stable within skeletal muscle during exercise (Mahoney et al., 2004), and was stable between the time points (data not shown). The threshold cycle (Ct) values of the target gene were normalized to Ct values of the internal control GAPDH, and final results were calculated as relative expression against the standard curve as described previously (Holloway et al., 2017). Statistical analysis for all mRNA data was performed on the delta Ct values. The baseline muscle biopsy was given a value of 1, and fold changes at 1 h post RE and 3 h post RE were calculated for figure presentation.

**Table 3:** Primers used for qPCR.

**Gene**	**Forward primer**	**Reverse primer**	**Amplicon size**
HBEGF	ATCGTGGGGCTTCTCATGTTT	TTAGTCATGCCCAACTTCACTTT	86
FOXO3A	CGGACAAACGGCTCACTCT	GGACCCGCATGAATCGACTAT	150
IGF-1	GCTCTTCAGTTCGTGTGTGGA	GCCTCCTTAGATCACAGCTCC	133
INSIG-1	CCTGGCATCATCGCCTGTT	AGAGTGACATTCCTCTGGATCTG	103
MAFbx	GCCTTTGTGCCTACAACTGAA	CTGCCCTTTGTCTGACAGAAT	187
MURF1	CTTCCAGGCTGCAAATCCCTA	ACACTCCGTGACGATCCATGA	116
Myostatin	TCCTCAGTAAACTTCGTCTGGA	CTGCTGTCATCCCTCTGGA	127
PDK4	GGAGCATTTCTCGCGCTACA	ACAGGCAATTCTTGTCGCAAA	117
PGC-1α	TCTGAGTCTGTATGGAGTGACAT	CCAAGTCGTTCACATCTAGTTCA	112
SIRT1	TAGCCTTGTCAGATAAGGAAGGA	ACAGCTTCACAGTCAACTTTGT	160
VCP	CAAACAGAAGAACCGTCCCAA	TCACCTCGGAACAACTGCAAT	114

### Statistics

Data was analyzed using a two-way repeated measures ANOVA (two factor, time x treatment) from IBM SPSS version 23 statistical software (IBM, Armonk, NY). When a main effect of condition or time or interaction was identified, a pairwise multiple comparison with a Bonferroni correction was done to identify differences. Statistical significance was set at the *P* < 0.05 level, and values were expressed as means ± SEM or different when indicated.

## Results

### Endurance and resistance exercise performance

Two subjects performed the endurance exercise sessions with a reduced workload. For one subject the workload was reduced with 20 W after 20 min whereas for the other subject the workload was reduced with 20 W after 30 min. There was no further reduction in workload during the remaining part of the endurance session. Additionally, both subjects were able to repeat this on the second experimental day. All 13 subjects performed the resistance exercise training with exactly the same amount of work on the 2 experimental days.

### Muscle glycogen

**Figure 2:**
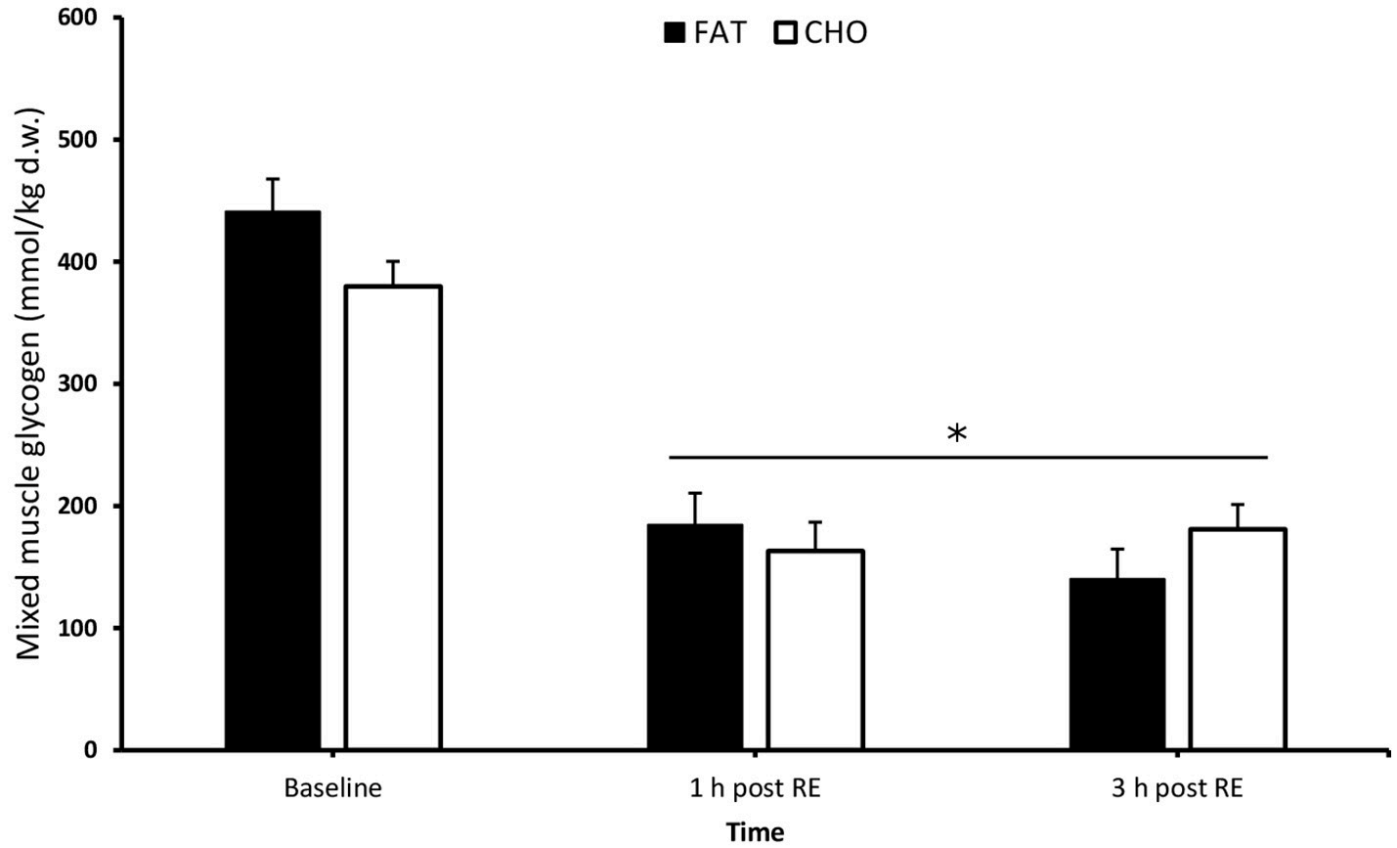
Mixed muscle glycogen at rest and 1 and 3 h after 5 sets of 8 repetitions for both leg press and leg extensions at 80% 1RM (1, 2, and 3 h RE) Values are mean ± SEM. *Significantly different (*P* < 0.05) vs. baseline. CHO, open bars; FAT, filled bars.

There were no differences in baseline muscle glycogen between the conditions [CHO 380 mmol/kg dry weight (dw) vs. FAT 441 mmol/kg dw; *P* > 0.05 Figure 2]. As a result of exercise, muscle glycogen was significantly reduced compared to baseline in both conditions at 1 and 3 h post-resistance exercise (*P* < 0.05) (carbohydrate 163 and 181 mmol/kg dw; fat 185 and 140 mmol/kg dw), without any significant differences between the conditions (*P* > 0.05).

### Free fatty acids, glucose, and insulin

**Figure 3:**
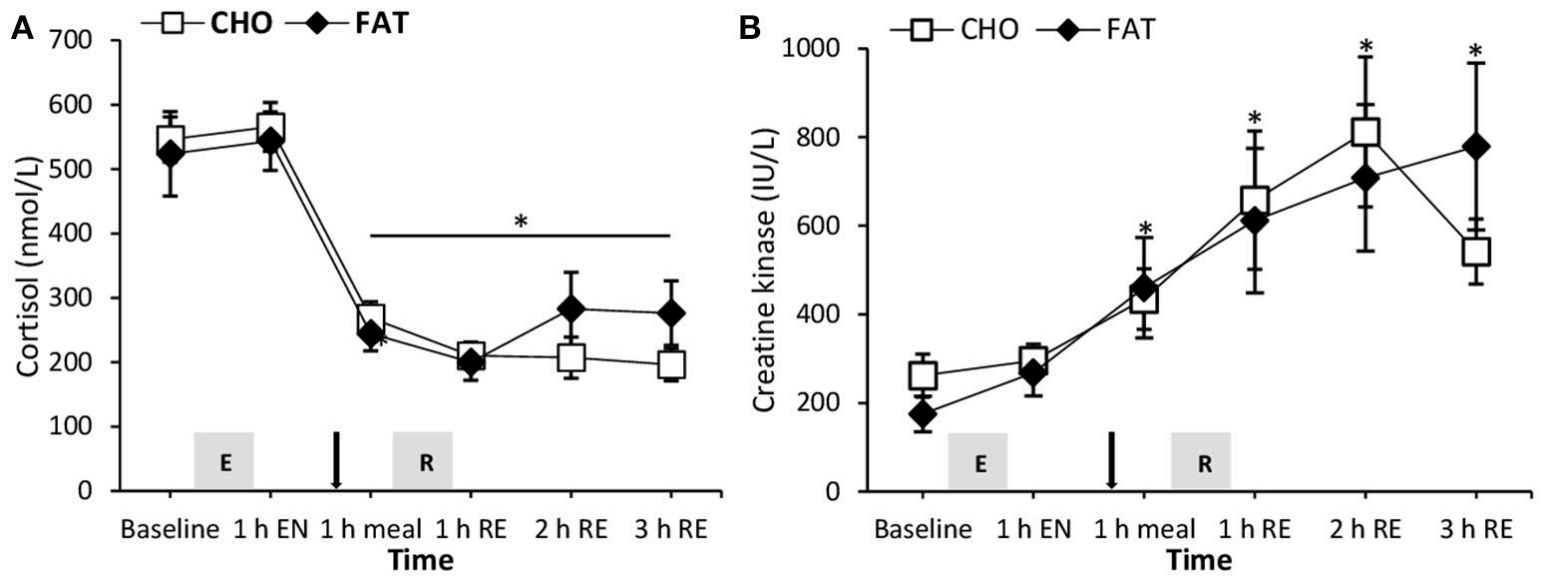
Plasma cortisol **(A)** and creatine kinase **(B)** at rest and 1 h after 90-min cycling 70% VO_2max_ (1 h EN), 1 h post-meal (1 h meal) and 1, 2, and 3 h after 5 sets of 8 repetitions for both leg press and leg extensions at 80% 1RM (1, 2, and 3 h RE) Values are mean ± SEM. *Significantly different (*P* < 0.05) vs. baseline. Gray box with E, endurance exercise bout; with R, resistance exercise bout; black arrow, mixed meal.

One hour post-meal, plasma free fatty acid concentration was higher in the FAT condition compared with the carbohydrate condition and this effect remained significant after 1 and 2 h post-resistance exercise (*P* < 0.05) (Figure 3). Both glucose and insulin in the fat condition were significantly lower at 1 h post-meal, and at 1, 2, and 3 h post-resistance exercise compared with the carbohydrate condition (*P* < 0.05) (Figure 3).

### Cortisol and creatine kinase

**Figure 4:**
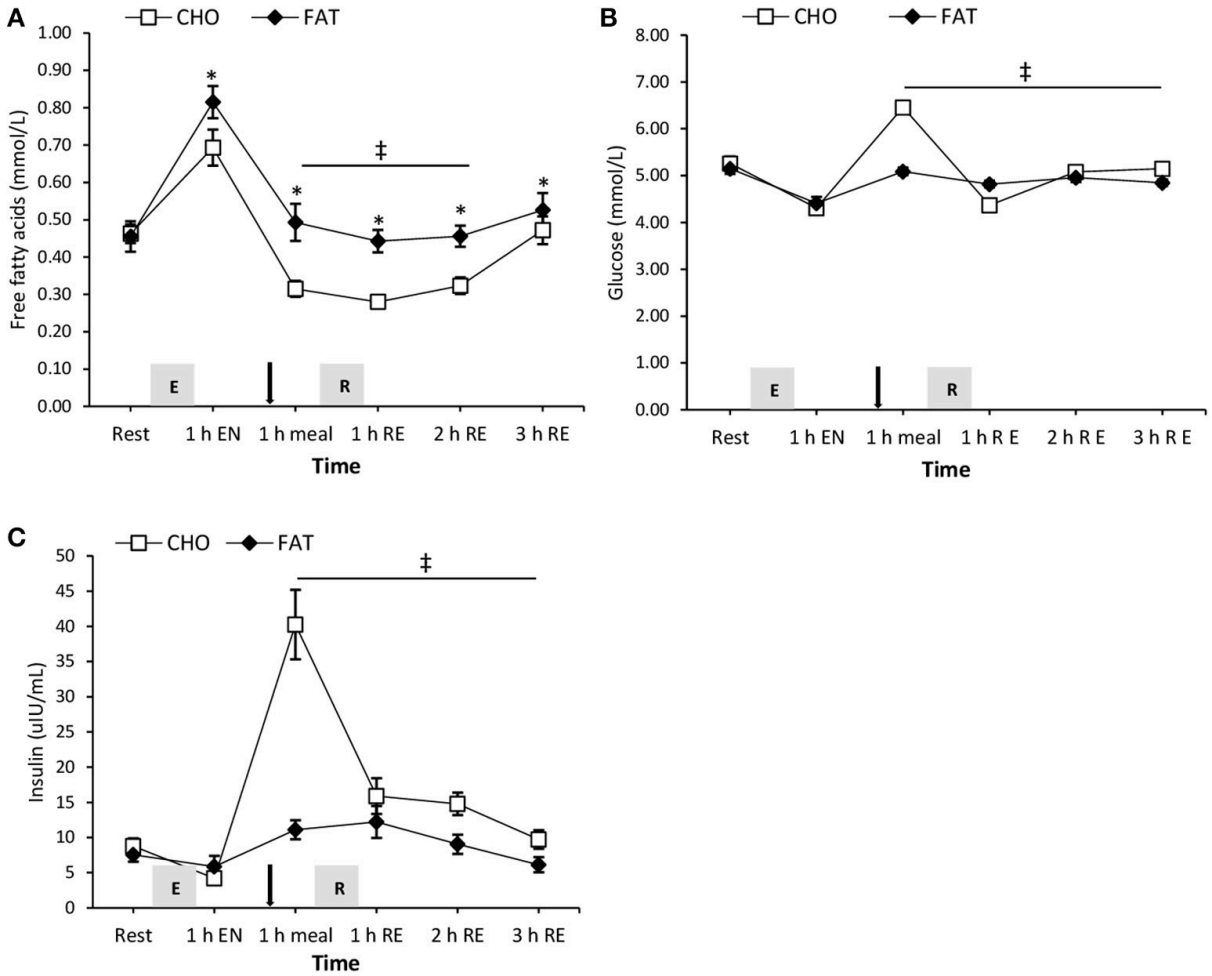
Plasma free fatty acids **(A)**, glucose **(B)**, and insulin **(C)** at rest and 1 h after 90-min cycling 70% VO_2max_ (1 h EN), 1 h post-meal (1 h meal) and 1, 2, and 3 h after 5 sets of 8 repetitions for both leg press and leg extensions at 80% 1RM (1, 2, and 3 h RE) Values are mean ± SEM. *Significantly different (*P* < 0.05) vs. baseline. ^‡^Significantly different (*P* < 0.05) between conditions (interaction). Gray box with E, endurance exercise bout; with R, resistance exercise bout; black arrow, mixed meal.

Both plasma creatine kinase and cortisol levels did not show any difference between the CHO and FAT condition (Figure 4). Compared with baseline and 1 h post-endurance exercise cortisol plasma levels were lower at all other time points during the day (*P* < 0.05). Plasma creatine kinase levels were higher at all-time points compared with baseline in both the CHO and FAT condition.

### Skeletal muscle gene expression

#### Substrate metabolism

**Figure 5:**
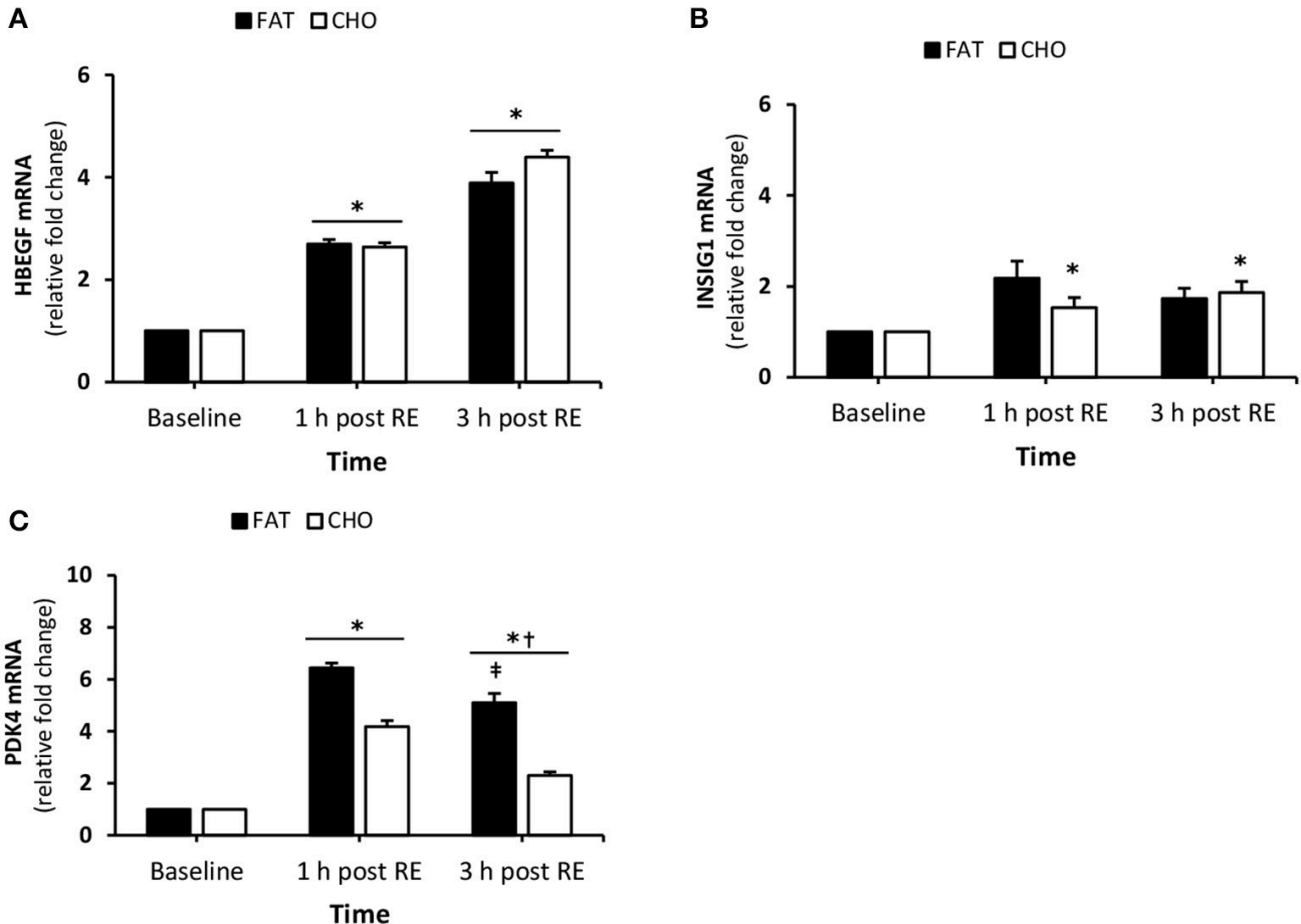
(Substrate metabolism) HBEGF **(A)**, INSIG1 **(B)**, and PDK4 **(C)** mRNA quantity levels at rest and 1 and 3 h post RE. Values are mean ± SEM. *Significantly different (*P* < 0.05) vs. baseline. ^†^Significantly different (*P* < 0.05) compared to previous time point. ^‡^Significantly different (*P* < 0.05) between conditions (interaction). CHO, open bars; FAT, filled bars.

There was a significant increase in HBEGF mRNA quantity at 1 and 3 h post-resistance exercise compared to baseline (*P* < 0.05), while no differences were observed between the treatments (Figure 5). PDK4 mRNA was increased 1 h post-resistance exercise (*P* < 0.05) in both conditions and significantly reduced at 3 h post-resistance exercise (Figure 5C). Additionally, at 3 h post-resistance PDK4 mRNA was higher in the FAT condition when compared with CHO (*P* < 0.05). The expression of INSIG1 increased at 1 h post-resistance exercise in the FAT group, whereas the levels in the CHO group increased 3 h post-resistance exercise compared to baseline (Figure 5B).

#### Proteolytic genes

**Figure 6:**
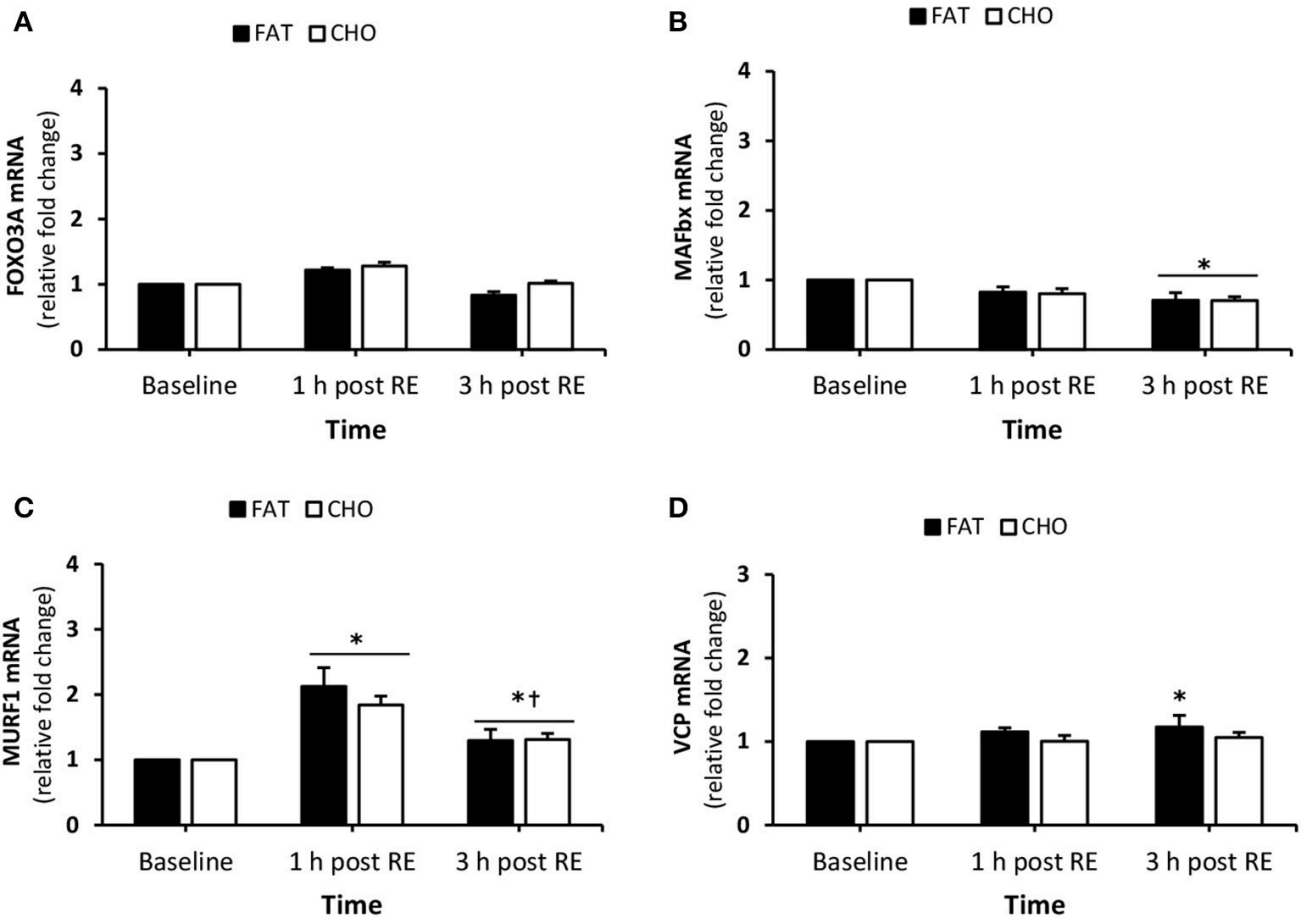
(Protein degradation) FOXO3A **(A)**, MAFbx **(B)**, MURF1 **(C)**, VCP **(D)** mRNA quantity levels at rest and 1 and 3 h post RE. Values are mean ± SEM. *Significantly different (*P* < 0.05) vs. baseline. ^†^Significantly different (*P* < 0.05) compared to previous time point. CHO, open bars; FAT, filled bars.

FOXO3A mRNA quantity did not change over the course of the day (Figure 6A). There was a reduction in both the CHO and FAT condition in MAFbx mRNA response at 3 h post-resistance resistance (*P* < 0.05) compared to baseline, however, no differences were found between the CHO and FAT condition at any time point (Figure 6B). MURF1 mRNA quantity increased in both conditions 1 and 3 h post-resistance exercise (*P* < 0.05) compared to baseline (Figure 6C). In addition MURF1 mRNA quantity decreased at 3 h post-resistance exercise when compared with 1 h post-resistance exercise (*P* < 0.05). No differences in MURF1 mRNA quantity was found between the FAT and CHO condition during the experimental days. VCP mRNA quantity was slightly higher at 3 h post-resistance exercise in the FAT group (*P* < 0.05) (Figure 6D).

#### Mitochondrial biogenesis

**Figure 7:**
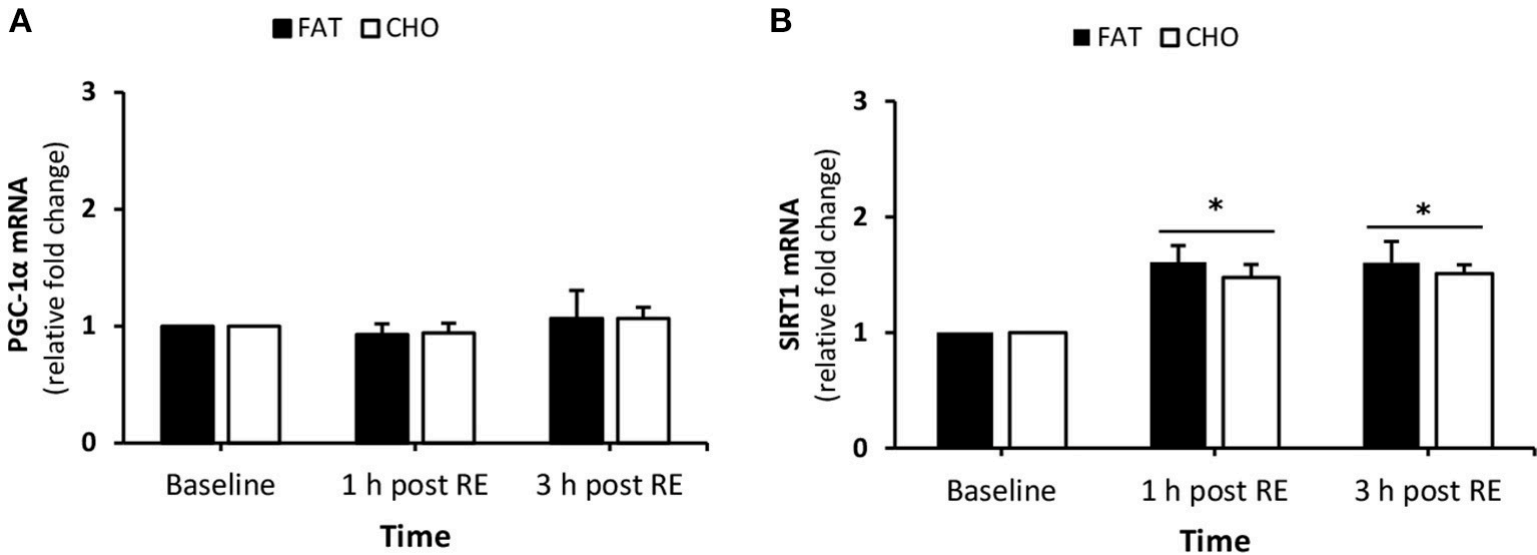
(Mitochondrial biogenesis) PGC-1a **(A)** and SIRT1 **(B)** mRNA quantity levels at rest and 1 and 3 h post RE. Values are mean ± SEM. *Significantly different (*P* < 0.05) vs. baseline. CHO, open bars; FAT, filled bars.

There was no effect of exercise nor nutrition on PGC-1α mRNA quantity (Figure 7A). In contrast, SIRT1 mRNA levels increased at 1 and 3 h post-resistance exercise compared with baseline, no differences were found between the CHO and FAT condition in SIRT1 mRNA quantity (Figure 7B).

#### IFG-1 and myostatin

**Figure 8:**
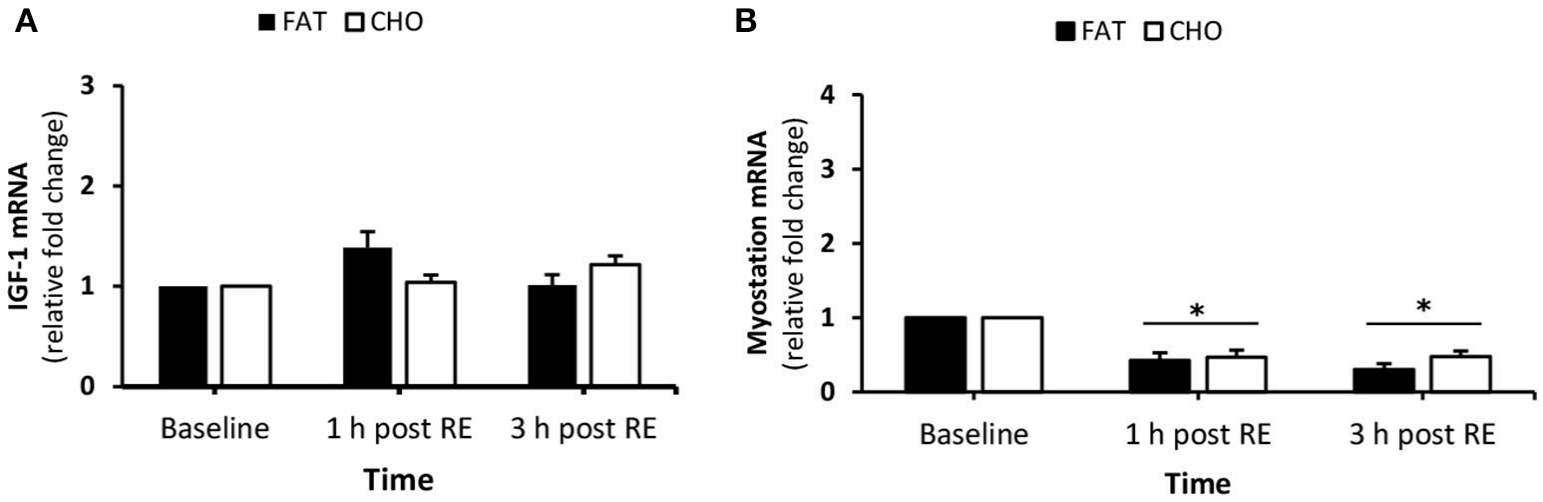
IGF-1 **(A)** and myostatin **(B)** mRNA quantity levels at rest and 1 and 3 h post RE. Values are mean ± SEM. *Significantly different (*P* < 0.05) vs. baseline. CHO, open bars; FAT, filled bars.

IGF-1 mRNA expression levels remained unchanged over the course of the day (Figure 8A). Myostatin mRNA decreased at 1 and 3 h post-resistance compared with baseline (*P* < 0.05) without any differences between the nutritional conditions (Figure 8B).

## Discussion

The gene expression profiles we investigated post-resistance exercise are of interest as they may play a role in mitochondrial biogenesis, protein degradation, and substrate metabolism. When compared with the CHO-rich meal condition, we observed an increase in PDK4 mRNA quantity suggesting that pre-exercise FAT meal in combination with the glycogen depletion potentially induced a shift in fuel selection from carbohydrate to fat in the post-resistance exercise period. However, we observed that the genes with a proposed role in muscle adaptation, responded as a result of exercise without any differences between the CHO and FAT condition. Therefore, we suggest that the acute post-resistance exercise response of genes involved in muscle remodeling is not affected by a pre-resistance exercise mixed meal containing different amounts of carbohydrates and fat.

Since athletes commonly combine divergent exercise sessions on the same day, we decided to include both endurance and resistance exercise on the same day in our protocol. Additionally, it has been recently proposed that, when combining divergent exercise sessions within the same day, the endurance session should be performed in the morning in the fasted state, with ample protein ingestion, while the afternoon resistance exercise session should be conducted only after carbohydrate replenishment with adequate post-resistance exercise protein ingestion (Perez-Schindler et al., 2015). With our study we aimed to evaluate the effect of a pre-resistance exercise isocaloric (~3200 kJ) mixed meal containing different amounts of carbohydrates and fat, on post-resistance exercise gene expression after glycogen depleting exercise earlier that day.

### Skeletal muscle glycogen

Our subjects performed an endurance exercise bout (90 min at ~70% VO_2max_) in the morning. It is generally assumed that full skeletal muscle glycogen stores are sufficient to fuel endurance type activities for~60–90 min (Bartlett et al., 2015). Therefore, it can be assumed that the endurance exercise resulted in a significant reduction of the skeletal muscle glycogen levels post-endurance exercise. Two hours after the endurance exercise bout subjects were fed with either the CHO or FAT meal. Maehlum and Hermansen (1978) showed that omitting carbohydrates in the acute post-exercise period is accompanied with a skeletal muscle glycogen synthesis rate of only 1–2 mmol/kg wet weight (w.w.) of muscle/h through gluconeogenesis (Maehlum and Hermansen, 1978). In contrast, when large amounts (~100 g of carbohydrates per hour) of carbohydrates are ingested in the post-exercise period the rate of liver and muscle glycogen synthesis can be up to 5–10 mmol/kg w.w./h (Burke et al., 2017). This increased rate of synthesis is primarily explained by insulin-mediated activation of glycogen synthase (Prats et al., 2009), exercise induced increase in insulin sensitivity (Richter et al., 1989), and enhanced muscle cell membrane permeability to glucose (Burke et al., 2017). With our approach, both at 1 and 3 h post-resistance exercise, skeletal muscle glycogen stores were reduced compared with baseline. Although the amount of carbohydrates in the CHO condition (~1.44 g·kg^−1^ carbohydrates) meets the recommendation to facilitate post-exercise muscle glycogen resynthesis (Moore, 2015), no differences in muscle glycogen were observed in the post-resistance exercise period when compared with the FAT condition (~0.26 g·kg^−1^ carbohydrates). Since we have no data on the muscle glycogen levels prior to the resistance exercise bout, it remains elusive to what extent the meals affected the muscle glycogen stores. It also remains unclear to what extent the resistance exercise bout further reduced muscle glycogen stores. While the mixed meal with different amounts of carbohydrates and fat did not result in differences in skeletal muscle glycogen levels, significant differences in plasma glucose, free fatty acids, and insulin were present. Therefore, we are convinced that the mixed meals resulted in a significant physiological difference with regards to macronutrient availability between the conditions.

### PDK4 mRNA

PDK4 phosphorylates pyruvate dehydrogenase enzyme, the first component of the pyruvate dehydrogenase complex that contributes to the conversion of pyruvate into acetyl-CoA altering fuel selection from carbohydrate to fat (Wang and Sahlin, 2012). Indeed, earlier work showed that PDK4 in human skeletal muscle is dependent on substrate availability rather than exercise-induced cellular perturbations (Cluberton et al., 2005; Psilander et al., 2013). Likewise, exercise performed with normal skeletal muscle glycogen levels show higher levels of PDK4 mRNA quantity compared with low glycogen levels (Psilander et al., 2013). Since we observed no differences in post-resistance exercise skeletal muscle glycogen levels between the nutritional conditions, the exogenous provision of carbohydrates ostensibly reduced PDK4 mRNA expression. Interestingly, our finding that exogenous carbohydrate ingestion reduces PDK4 expression is in line with earlier investigations where PDK4 mRNA expression was reduced when exogenous carbohydrate was provided (Cluberton et al., 2005) or when skeletal muscle glycogen were normal compared to low (Psilander et al., 2013).

### PGC-1α mRNA

PGC-1α has been proposed as the master regulator of mitochondrial biogenesis. Furthermore, PGC-1α exists in different isoforms, which may have different roles in training adaptation. For instance, PGC-1α4 has been implicated to play a role in the regulation of skeletal muscle hypertrophy (Ruas et al., 2012). Unexpectedly, PGC-1α mRNA quantity remained unchanged during the post-resistance exercise period. This was somewhat surprising since work by Camera et al. (2015) recently demonstrated that performing resistance exercise with low skeletal muscle glycogen levels amplifies intramuscular PGC-1α mRNA quantity when compared with normal glycogen levels (Camera et al., 2015). Notably, the time course of the muscle biopsies differed between the studies. Camera et al. (2015) took muscle biopsies 2 and 4 h post-resistance exercise and found enhanced expression of PGC-1α at the 4 h time point. We took biopsies 1 and 3 h post-resistance exercise and may therefore overlooked a potential effect of the intervention PGC-1α mRNA quantity. Furthermore, our subjects were provided we a mixed meal in the pre-resistance exercise period which makes is difficult to compare the divergent findings of the studies. Lastly, it has been reported that BCAA provision attenuates the resistance exercise induced elevation of PGC-1α4 mRNA, though, no effect of BCAA was found on PGC-1α1 and 2 mRNA (Samuelsson et al., 2016). Nevertheless, it may thus be possible that the post-exercise protein ingestion attenuated the PGC-1α mRNA response.

### Gene expression of proteolytic genes

We also analyzed a set of genes with a proposed role in protein degradation (FOXO3A, MAFbx, MURF1, Myostatin, and VCP). Myostatin mRNA quantity decreased post-resistance exercise, which is in accordance with findings of others (Camera et al., 2012). Muscle catabolism is a fundamental process of muscle remodeling ensuring that damaged proteins or misfolded proteins during exercise are adequately removed from the cell (Nader et al., 2014). As expected, mRNA involved in both ubiquitin proteasome and autophagy-mediated protein degradation systems responded as a result of exercise, however without any effect of the pre-resistance exercise mixed meal. We found a higher plasma insulin response in the carbohydrate group. According to Abdulla et al. (2016) insulin reduces protein degradation rates thereby facilitating an overall net protein balance (Abdulla et al., 2016). Mechanistically, insulin activates the PI3K-Akt pathway by binding to its transmembrane insulin receptor on the sarcolemma and initiates translocation of the proteins to the cell membrane (Marcotte et al., 2015). Earlier work in animals (Mikura et al., 2009) and C2C12 cell lines (Stitt et al., 2004; Latres et al., 2005) demonstrated that Akt inhibits the expression of FOXO3A mRNA, a transcription factor implicated with gene expression with the ubiquitin proteasome pathway. In our study, the mixed meal with the higher amount of carbohydrates resulted in higher insulin response post-resistance exercise, however, this effect did not translate into a difference in FOXO3A mRNA response between the CHO and FAT condition. The latter could be explained by the theory that the insulin mediated reduction in protein breakdown is more potent when amino acid availability is scarce (Bird et al., 2006a,b; Abdulla et al., 2016). This was not the case in our study since our subjects were provided with adequate protein ingestion post-resistance exercise (Morton et al., 2015). Other reports confirm that when adequate protein is provided post-resistance exercise, the insulinogenic response seems to be redundant (Staples et al., 2011; Morton et al., 2015).

## Conclusion

In summary, resistance exercise with different carbohydrate/fat availability but ample protein provision did in general not influence intramuscular gene expression. Post-resistance exercise PDK4 mRNA quantity was higher in the CHO condition compared with the FAT condition suggesting a potential shift toward glucose oxidation. Furthermore, it appears that carbohydrate replenishment in between endurance and resistance exercise did not influence gene expression involved in the adaptive response after a subsequent bout of resistance exercise. Our findings support the view that pre-resistance exercise carbohydrate availability does not affect acute transcriptional responses associated with muscle recovery to resistance exercise.

## Author contributions

MM, MH, and PK designed the methodology of the study. PK wrote the manuscript. PK performed the data acquisition. MM, MH, and JW contributed substantially by giving insightful comments and suggestions during the creation of the manuscript.

### Conflict of interest statement

The authors declare that the research was conducted in the absence of any commercial or financial relationships that could be construed as a potential conflict of interest.

## Abbreviations

FOXO3A: Forkhead box O3
HBEGF: Heparin-binding EGF-like growth factor
IGF-1: Insulin-like growth factor 1
INSIG1: Insulin induced gene 1
MAFbx: Transciption factor MafB
MURF1: E3 ubiquitin-protein ligase
PDK4: Pyruvate dehydrogenase kinase isozyme 4
PGC-1α: Peroxisome proliferator-activated receptor gamma coactivator 1-alpha
SIRT1: NAD-dependent deacetylase sirtuin-1
VCP: Valosin-containing protein.
